# Botulinum Toxin Is Effective in the Management of Neurogenic Dysphagia. Clinical-Electrophysiological Findings and Tips on Safety in Different Neurological Disorders

**DOI:** 10.3389/fphar.2017.00080

**Published:** 2017-02-22

**Authors:** Enrico Alfonsi, Domenico A. Restivo, Giuseppe Cosentino, Roberto De Icco, Giulia Bertino, Antonio Schindler, Massimiliano Todisco, Mauro Fresia, Andrea Cortese, Paolo Prunetti, Matteo C. Ramusino, Arrigo Moglia, Giorgio Sandrini, Cristina Tassorelli

**Affiliations:** ^1^Department of Neurophysiopathology, National Neurological Institute “C. Mondino” (IRCCS)Pavia, Italy; ^2^Department of Neurology, Garibaldi HospitalCatania, Italy; ^3^Department of Biomedicine and Clinical Neuroscience, University of PalermoPalermo, Italy; ^4^Department of Brain and Behavioral Sciences, University of PaviaPavia, Italy; ^5^Department of Otolaryngology, San Matteo Hospital, University of PaviaPavia, Italy; ^6^Department of Clinical Sciences “Luigi Sacco”, University of MilanMilan, Italy

**Keywords:** botulinum toxin, neurogenic dysphagia, electrophysiological study of swallowing, cricopharyngeal muscle, upper esophageal sphincter dysmotility

## Abstract

**Background and Aims:** Neurogenic dysphagia linked to failed relaxation of the upper esophageal sphincter (UES) can be treated by injecting botulinum toxin (BTX) into the cricopharyngeal (CP) muscle. We compared the effects of this treatment in different neurological disorders with dysphagia, to evaluate its efficacy over time including the response to a second injection.

**Materials and Methods:** Sixty-seven patients with neurogenic dysphagia associated with incomplete or absent opening of the UES (24 with brainstem or hemispheric stroke, 21 with parkinsonian syndromes, 12 with multiple sclerosis, and 10 with spastic-dystonic syndromes secondary to post-traumatic encephalopathy) were treated with the injection of IncobotulinumtoxinA (dose 15–20 U) into the CP muscle under electromyographic guidance. The patients were assessed at baseline and after the first and second treatment through clinical evaluation and fiberoptic endoscopy of swallowing, while their dysphagia was quantified using the Dysphagia Outcome and Severity Scale (DOSS). An electrokinesiographic/electromyographic study of swallowing was performed at baseline.

**Results**: Most patients responded to the first BTX treatment: 35 patients (52.2%) were classified as high responders (DOSS score increase >2 levels), while other 19 patients (28.4%) were low responders (DOSS score increase of ≤2 levels). The effect of the first treatment usually lasted longer than 4 months (67%), and in some cases up to a year. The treatment efficacy remained high also after the second injection: 31 patients (46.3%) qualified as high responders and other 22 patients (32.8%) showed a low response. Only in the parkinsonian syndromes group we observed a reduction in the percentage of high responders as compared with the first treatment. Side effects were mostly mild and reported in non-responders following the first injection. A severe side effect, consisting of ingestion pneumonia, was observed following the second BTX injection in two patients who had both been non-responders to the first. Non-responders were characterized electromyographically by higher values of the oropharyngeal interval.

**Conclusion:** These findings confirm the effectiveness of IncobotulinumtoxinA injection in the treatment of neurogenic dysphagia due to hyperactivity and relaxation failure of the UES. Caution should be used as regards, the re-injection in non-responders to the first treatment.

## Introduction

Neurogenic dysphagia associated with failed relaxation of the upper esophageal sphincter (UES) has been observed in patients suffering from different types of neurological disease, such as parkinsonisms, multiple sclerosis (MS) and stroke (Regan et al., 2014). The absent relaxation of the cricopharyngeal (CP) muscle during bolus swallowing prevents the UES from opening; consequently, the bolus cannot progress into the esophagus. This may result in penetration or aspiration of ingested food into the airways.

Botulinum toxin (BTX) is one of the most effective options for the treatment of neurological disorders characterized by dysfunction of striated muscle (hyperactivity, dyskinesia, or prolonged spasms) (Orsini et al., 2015; Sivaraman Nair, 2016). Several reports in the literature indicate that neurogenic dysphagia associated with UES spasms or dyskinesia can be effectively treated by injecting BTX into the CP muscle (Ahsan et al., 2000; Alberty et al., 2000; Haapaniemi et al., 2001; Moerman, 2006; Krause et al., 2008; Terré et al., 2008, 2013; Alfonsi et al., 2010; Restivo et al., 2013; Regan et al., 2014). However, most of these reports are case series formed by a low number of patients, while randomized control trials are lacking. Moreover, because of different methodological approaches, study designs and outcome measures, the results obtained by different authors are not strictly comparable. Indeed patients’ selection criteria vary greatly from study to study, and the same is also true for follow-up times: some authors have focused only on short-term safety and efficacy of BTX treatment, while others have investigated long-term effects (Ahsan et al., 2000; Haapaniemi et al., 2001; Shaw and Searl, 2001; Zaninotto et al., 2004; Terré et al., 2008, 2013; Woisard-Bassols et al., 2013).

Oropharyngeal swallowing, in its different phases, can be investigated using a combined electrokinesigraphic/electromyographic method that includes multichannel recording of the EMG activity of the submental/suprahyoid muscle complex and the CP muscle (the main portion of the UES), and mechanographic recording of the kinematic aspects of the pharyngeal-laryngeal structures occurring during the pharyngeal phase of swallowing.

In a previous electrokinesiographic/electromyographic study of swallowing (EES), we showed that BTX injected into the hyperactive CP muscle of patients with dysphagia can restore the brief disappearance of EMG activity (inhibitory pause) that normally occurs in this muscle during the pharyngeal phase of swallowing (Alfonsi et al., 2010). We also identified specific alterations in the electrophysiological patterns of oropharyngeal swallowing, some of which are frequently associated with inefficacy of BTX treatment. A recent Cochrane review on the use of BTX in neurogenic dysphagia concluded that there is still insufficient evidence to inform clinical practice (Regan et al., 2014). Indeed, although it has been suggested that there may be different reasons for failure of BTX injection into the CP muscle to treat neurogenic dysphagia (Alfonsi et al., 2010), the factors that may influence the therapeutic response or predict the risk of side effects, thus contraindicating the treatment, are still poorly understood (Regan et al., 2014). In addition, there are currently no studies comparing the effectiveness of this BTX treatment in different neurological disorders with dysphagia associated with failed UES relaxation.

Therefore, the main aims of this study were: (1) to analyze the safety and effectiveness of BTX treatment for neurogenic dysphagia in different neurological diseases characterized by abnormally reduced or absent relaxation of the CP muscle, (2) to evaluate consistency of effectiveness over two subsequent injections, and 3) to search for reliable EES indicators of responsiveness to the BTX treatment.

## Materials and Methods

### Ethics Statement

The study protocol was approved by the Ethics Committee of the C. Mondino National Neurological Institute. All the patients gave their informed consent to all the study procedures.

### Subjects

In this prospective, observational study, we enrolled 67 patients consecutively hospitalized at our institute suffering from neurogenic dysphagia associated with incomplete or absent opening of the UES due to hypertonia and/or dyskinesia of the CP muscle. The main characteristics of the patients – 41 men and 26 women (mean age ± SD: 63.5 ± 13.5 years; age range: 43–87 years) – are summarized in **Table 1**. Twenty-four of them had suffered a stroke more than six months previously: the lesion was located in the lateral medullary region (Wallenberg syndrome) in 14 subjects, while the remaining 10 had a lesion in one hemisphere. Twenty-one subjects had parkinsonian syndromes; of these, 12 had idiopathic Parkinson’s disease (PD) diagnosed according to the U.K. Parkinson’s Disease Brain Bank criteria (Hughes et al., 1992), and were examined during the “on” phase of the levodopa response cycle, five had multiple system atrophy, parkinsonian variant (MSA-P), diagnosed according to the criteria of the ‘2nd Consensus Statement on the diagnosis of multiple system atrophy’ (Gilman et al., 2008), and four had progressive supranuclear palsy (PSP) diagnosed as per the criteria proposed by NINDS-SPSP (Litvan et al., 1996). Twelve patients had MS: nine with the secondary progressive form, and three with the primary progressive form diagnosed according to McDonald et al. (2001). The remaining 10 patients had stabilized symptoms of spastic dystonia due to post-traumatic encephalopathy.

**Table 1:** Botulinum toxin (BTX) injection into the cricopharyngeal (CP) muscle in neurogenic dysphagia. Epidemiologic and clinical aspects of the patients.

		Total	Parkinsonian syndromes (*N* = 21)			Stroke (*N* = 24)		Post-traumatic encephalopathy	Multiple Sclerosis
			PD	MSA-P	PSP	Cerebral lesion	Brainstem lesion		
Number of cases		67	12	5	4	10	14	10	12
Age (years) (mean ±*SD*)		63.5 ± 13.5	69.0 ± 5.9	67.8 ± 7.0	68.0 ± 7.1	67.9 ± 8.8	65.0 ± 10.3	55.3 ± 21.1	48.7 ± 13.7
Sex (Male/Female)		41/26	4/8	3/2	2/2	9/5	9/1	8/2	3/9
Time post-diagnosis/lesion (years) (mean ±*SD*)		–	9.7 ± 2.1	4.5 ± 0.7	6.5 ± 2.1	4.8 ± 1.9	5.4 ± 2.6	7.2 ± 3.8	9.2 ± 6.2
Severity of dysphagia (DOSS)	Mild (score 5–4)	20 (29.8%)	8 (66.7%)	–	–	6 (60.0%)	–	–	6 (50.0%)
	Moderate (score 3–2)	30 (44.8%)	3 (23.0%)	2 (40.0%)	2 (50.0%)	4 (40.0%)	7 (50.0%)	8 (80.0%)	4 (33.3%)
	Severe (score 1)	17 (25.4%)	1 (8.3%)	3 (60.0%)	2 (50.0%)	–	7 (50.0%)	2 (20.0%)	2 (16.7%)

*PD, idiopathic Parkinson’s disease; MSA-P, multiple system atrophy, parkinsonian variant; PSP, progressive supranuclear palsy; DOSS, Dystonia Outcome and Severity Scale.*

### Methods

All the patients underwent bedside examination (BSE) and fiberoptic endoscopic evaluation of swallowing (FEES). Based on findings from BSE and FEES, dysphagia was graded according to the Dysphagia Outcome and Severity Scale (DOSS) (O’Neil et al., 1999), which allowed the identification of the following three levels of dysphagia: scores 5 and 4 = mild dysphagia; scores 3 and 2 = moderate dysphagia; score 1 = severe dysphagia.

**FIGURE 1:**
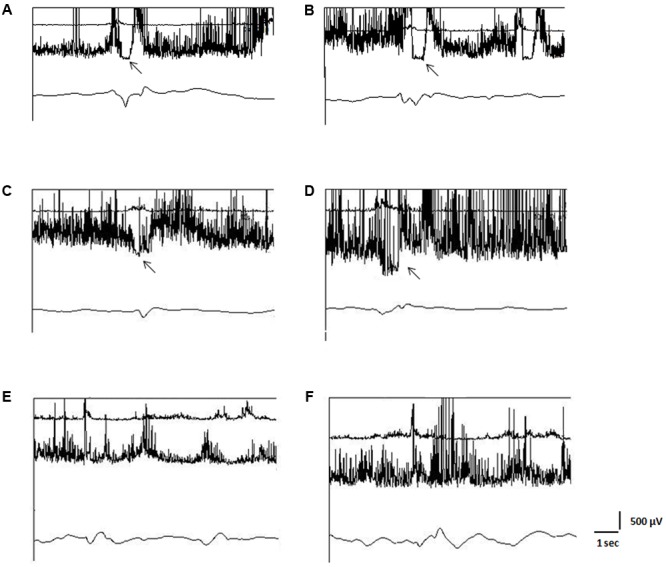
**Electrokinesiographic/Elecromyographic study of swallowing (EES).** In each panel the upper trace represents the surface EMG activity of the suprahyoid/submental muscle complex, the middle trace the needle-EMG activity of the cricopharyngeal muscle (CP), and the lower trace is the mechanogram obtained from a piezoelectric transducer applied to the skin over the cricothyroid membrane. In **(A,B)**: traces recorded from a normal subject. The EMG pause of the CP muscle is evident on both sides (**A**: left; **B**: right). In **(C,D)**: patient with stroke lesion of the left hemisphere and paresis on the right side. The EMG pause of the CP muscle is bilaterally reduced, more in the right side **(D)** as compared to the left one **(C)**. Botulinum toxin (BTX) was injected into the right side of the CP muscle. In **(E,F)**: patient with spastic-dystonic syndrome in post-traumatic encephalopathy. EMG pause of the CP muscle is absent on both sides. Asymmetric tonic EMG activity of the CP muscle can be observed, being the EMG amplitude in the right side **(F)** greater than in the left one **(E)**. BTX was injected into the right side of the CP muscle. Arrows indicate pause of the EMG activity of the CP muscle.

Fiberoptic endoscopic evaluation of swallowing provides static and dynamic evaluation of the structures involved in the swallowing act. For the evaluation of these aspects, FEES compares well with videofluoroscopy (VFS) (Horner et al., 1993; Doggett et al., 2001). However impaired opening of UES may be only indirectly inferred with FEES when residual material is viewed in the pharyngeal recesses immediately after the “white out” phase, during which larynx adducts, pharynx constricts and epiglottis falls over the opening to the upper airway (Hiss and Postma, 2003). For this reason, FEES cannot be used to establish whether poor or absent UES opening is due to impaired relaxation of the CP muscle or to a mechanical dysfunction leading to impaired elevation and anterior movement of the laryngeal-pharyngeal structures during swallowing (Lang and Shaker, 2000; Sivarao and Goyal, 2000). Therefore, we also performed an EES to identify patients in whom incomplete or absent opening of the UES was due to a relaxation deficit of the CP muscle. EES was performed according to the methods of Ertekin et al. (1995) and Alfonsi et al. (2007), and it involved simultaneous, three-channel recording (**Figure 1**). The first channel recorded the EMG activity of the suprahyoid/submental muscles using surface electrodes. This activity marks the beginning of the propulsive action of the suprahyoid/submental muscles in the oral and pharyngeal phases of swallowing. The second channel recorded the EMG activity of the CP muscle using a concentric needle inserted through the skin at the level of the cricoid cartilage, about 1.5 cm posterior to its palpable lateral border, in the posteromedial direction. The CP muscle is a major contributor to the functional area known as the UES and at rest it shows tonic EMG activity related to its function as a sphincter (Lang and Shaker, 2000); during the pharyngeal phase of swallowing this EMG activity disappears completely for a brief time (inhibitory pause). During this pause, the amplitude of the EMG signal from this muscle falls below 50 μV (Alfonsi et al., 2010). The duration of the EMG pause of the CP muscle (CPEMG-PD) depends on the consistency and volume of the bolus (Lang and Shaker, 2000; Sivarao and Goyal, 2000) and ranges from 150 to 700 ms (mean ± SD: 470 ± 160 ms) according to the reference values obtained in our laboratory in a population of 40 healthy subjects (25M/15F, mean age ± SD: 58.3 ± 15.1 years; age range: 45–80 years) (Alfonsi et al., 2007, 2013). EMG signals from the first and second channels were rectified and bandpass filtered between 100 and 2 KHz. The third channel recorded the signal obtained from a piezoelectric transducer applied to the skin over the cricothyroid membrane. This transducer consists in a rectangular strip with a triangular rubber button in the center, which was applied to the skin over the cricothyroid membrane and kept in place by adhesive tape wrapped around the neck. It showed a linear force-to-signal ratio for forces ranging from 0.1 to 300 g. Its signals were bandpass filtered between 0.01 and 30 Hz. This device is able to record the moment of epiglottis elevation/retroflection and also the moment of its return to the rest position.

For EES, each subject was examined while seated with the head held in a natural position; a small volume of water (3 ml) was infused directly into the oral cavity using a disposable syringe. This was done gradually (over a period of 2 s) in order to avoid squirting the water into the posterior pharynx. The subject was instructed to keep the bolus at mid-tongue level, and thereafter, on feeling the liquid to be in the right position, to swallow it. We deliberately used a small fluid bolus (3 ml) to test swallowing activity because swallowing such a bolus requires a greater level of neural activation in individuals with neurological disorders (Kaas et al., 1983). In addition, given the risk of aspiration in dysphagic patients, this approach constituted a safety measure.

In addition to the above, two other electrophysiological parameters were evaluated in each patient: (1) the interval between the onset of the EMG activity of the submental/suprahyoid muscle (SHEMG) complex and the onset of the laryngopharyngeal mechanogram (LPM) measured in ms (I-SHEMG-LMP) (normal mean values ± SD: 200 ± 84 ms, range: 55–370 ms, obtained as for CPEMG-PD); (2) the duration of the SHEMG (SHEMG-D) measured in ms (normal mean values ± SD: 1126 ± 264 ms, range: 700–1820 ms, obtained as for CPEMG-PD). We decided to evaluate these parameters because I-SHEMG-LMP values greater than 300 ms, and SHEMG-D values greater than 1680 ms or below 1000 ms, recorded in a 3 cc liquid bolus swallowing task, were previously found to negatively influence the response to the BTX treatment in patients with CP muscle hyperactivity (Alfonsi et al., 2010).

Failure of relaxation was defined as complete when the EMG pause of the CP muscle (EMG-CP-pause) was absent, i.e., the EMG signal never fell below 50 μV (Alfonsi et al., 2010) or when the EMG signal fell below the lower limit of the normal CPEMG-PD range (less than 150 ms). Patients were considered eligible for BTX injection only if EES confirmed the failure of CP relaxation during the pharyngeal phase of swallowing.

The injection procedure was carried out using a needle electrode cannula (monopolar electrode), while monitoring the EMG activity of the muscle. This electrode was inserted in the neck at the level of the cricoid cartilage in the manner described above; a surface electrode (reference electrode) was applied ipsilaterally over the midline of the clavicle. The BTX injection was performed unilaterally in all patients because bilateral injection could have increased the risk of aspiration. Indeed, BTX not only favors CP muscle relaxation during the pharyngeal phase of swallowing, but it can also reduce the tonic muscle contraction, as shown in studies using esophageal manometry (Terré et al., 2008). Thus, loss of the sphincter activity of the CP muscle would increase the risk of food reflux from the esophagus, and consequent post-deglutition aspiration into the airways (Shaker and Hogan, 2000). A further reason for choosing unilateral injection was to reduce the effects of potential contamination of nearby muscles involved in vocal cord movement (the posterior and lateral cricoarytenoideus muscles) and pharyngeal peristalsis (the inferior constrictor muscle). Patients with unilateral spasticity, such as stroke patients, most of the patients with spastic-dystonic encephalopathy, and some of the patients with MS were injected with BTX on the paretic side, as the CP muscle tonic activity and impaired relaxation during swallowing were always greater on this side than contralaterally. In the other patients, the BTX injection was performed on the side showing greater CP muscle hyperactivity (i.e., greater mean amplitude of the tonic EMG activity at rest in a time of 5 s) (**Figure 1**).

In each patient, a 15 or 20 U dose of BTX (IncobotulinumtoxinA- Xeomin^®^; Merz, Frankfurt, Germany), diluted 5.0U/0.1 ml, was injected into the CP muscle on one side. The 15 U dose was used in patients in whom the baseline amplitude of the EMG signal from the CP muscle fell below 20 μV during the pharyngeal phase of swallowing, and the 20 U dose in those in whom it failed to drop below this level.

### Design of the Study

The study was conducted as a prospective observational investigation in which all patients with absent opening of the UES documented by FEES and an absent or incomplete EMG-CP-pause during the pharyngeal phase of swallowing underwent BTX injection into the CP muscle.

One month after BTX injection the patients were re-evaluated with BSE and FEES and arbitrarily classified as high responders, when the DOSS score increased by >2 levels (e.g., marked improvement of dysphagia from severe to mild), low responders when the DOSS score had increased by ≤2 levels (e.g., improvement of dysphagia from severe to moderate, or from moderate to mild), and non-responders when no score change was detected or DOSS score indicated a worsening of dysphagia. In each patient, the duration of the therapeutic effect of the first BTX injection was monitored by means of monthly clinical and FEES evaluations. For statistical purposes, the duration of BTX effect was stratified as follows: <2 months, 2–4 months, and >4 months. In responders, a second BTX injection was given when the DOSS score had returned to its baseline value. In non-responders a second BTX injection was administered one month after the first one.

Adverse events were carefully recorded at each clinical evaluation and were defined as mild when they did not interfere with normal daily living, moderate when they required medical treatment, and severe when hospitalization was required and/or life-threatening manifestations occurred. Regardless of the scheduled follow-up, all patients were advised to call or return to our Center if unexpected side effects occurred.

The primary outcome measure was the DOSS score change one month after injection, whereas the secondary outcome measure was the duration of the therapeutic effect of the first BTX injection.

### Statistical Analyses

Statistical analyses were performed using the Statistical Package for Social Sciences (SPSS) version 21.0 for Windows. Categorical variables were plotted in cross-tables and analyzed by means of the chi-square test. For the analysis of continuous variables, we first evaluated whether or not they showed a normal distribution, using a test for normality (Kolmogorov–Smirnov): SHEMG-D proved to be normally distributed, therefore differences between groups were tested with a parametric ANOVA, followed by a *post hoc* Bonferroni’s test; I-SHEMG-LMP was not normally distributed and so statistical analysis was performed by running a Kruskal–Wallis test followed by a Mann–Whitney test with Bonferroni’s correction. For each electrophysiological parameter, we considered the occurrence of values exceeding the normal limits, the normal range being taken to correspond to the 5th–95th percentile computed in a control group (reference values of our laboratory). The level of significance was set at 0.05 for each test.

## Results

**Table 1** describes the epidemiologic and clinical features of the study population at baseline. Seventeen of the 67 enrolled patients had severe dysphagia (7 with lateral medullary stroke, 6 with parkinsonian syndrome, 2 with post-traumatic encephalopathy, and 2 with MS), 30 had moderate dysphagia (7 with bulbar ischemia and 4 with unilateral hemispheric stroke, 7 with parkinsonian syndrome, 8 with post-traumatic encephalopathy, and 4 with MS), and the remaining 20 had mild dysphagia (6 with unilateral hemispheric stroke, 8 with parkinsonian syndrome, and 6 with MS). The clinical response to the first and second BTX injections into the CP muscle in this population is described in **Table 2**.

**Table 2:** Efficacy of BTX treatment in neurogenic dysphagia (1 month after BTX injection).

	Total	Parkinsonian syndromes			Stroke		Post-traumatic encephalopathy	Multiple Sclerosis
		PD	MSA-P	PSP	Cerebral lesion	Brainstem lesion		
Number of cases	67 (%)	12	5	4	10	14	10	12
**First injection of BTX**						
High responder	35 (52%)	6 (50.0%)	2 (40.0%)	1 (25.0%)	7 (70.0%)	4 (28.6%)	8 (80.0%)	7 (58.3%)
Low responder	19 (28%)	4 (33.3%)	2 (40.0%)	1 (25.0%)	1 (10.0%)	7 (50.0%)	2 (20.0%)	2 (16.7%)
Non-responder	13 (19%)	2 (16.7%)	1 (20.0%)	2 (50.0%)	2 (20.0%)	3 (21.4%)	0 (0.0%)	3 (25.0%)
**Second injection of BTX**						
High responder	31 (46.3%)	2 (16.7%)	0 (0.0%)	0 (0.0%)	4 (40.0%)	3 (21.4%)	10 (100%)	3 (25.0%)
Low responder	22 (32.8%)	7 (58.3%)	4 (80.0%)	2 (50.0%)	5 (50.0%)	8 (57.2%)	0 (0.0%)	5 (41.7%)
Non-responder	14 (20.9%)	3 (25.0%)	1 (20.0%)	2 (50.0%)	1 (10.0%)	3 (21.4%)	0 (0.0%)	4 (33.3%)

*First vs. second injection: χ^2^= 16.16, df = 4, *p* = 0.003. PD, idiopathic Parkinson’s disease; MSA-P, multiple system atrophy, parkinsonian variant; PSP, progressive supranuclear palsy.*

### Safety of the Treatment

After the first BTX inoculation, some drug-related mild side effects were observed in 10 of the 13 patients classified as non-responders (15% of total number of patients). In particular, in seven patients we observed a transient worsening of dysphagia for a period ranging from 1 week (in 2 patients with brainstem stroke) to 3 weeks (in 2 patients with PD, 1 patient with PSP, 1 with MSA-P, and 1 with previous lateral medullary stroke). In the other three patients, we observed transitory dysphonia (breathiness/whispering voice) lasting 1 week in one case (a patient with previous stroke in one hemisphere) and 2 weeks in the other two cases (1 patient with PD and 1 with PSP). In all cases the adverse effects did not require any dietary changes, medical treatment or hospitalization.

As regards, the second BTX injection, two of the non-responders (1 with MSA-P and 1 with PSP) showed, about one month after the treatment, a severe side effect consisting of ingestion pneumonia, which was associated with a worsening of their dysphagia. Both patients were hospitalized, treated with antibiotics, and recovered in a period of about 10 days. The side effect was considered possibly related to the drug, although, as both subjects were non-responders, we cannot exclude that it was simply related to the persistence/worsening of the underlying disease.

In addition, between one and three days after second injection, five patients (3 with PD, 1 with MSA-P, and 1 with previous bulbar stroke), all of whom non-responders, showed mild dysphonia (breathiness/whispering voice) lasting 1 week and not requiring any treatment.

### Effectiveness of the Treatment

**FIGURE 2:**
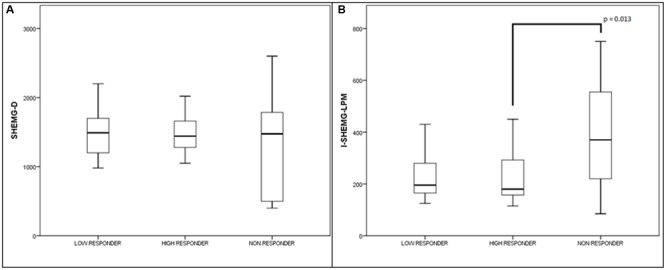
**High responders, low responders, and no-responders treated with injection of BTX into the CP muscle.** Values (median and interquartile range) of the electrophysiological parameters. **(A)** Duration of the EMG activity of suprahyoid /submental muscle complex (SHEMG-D), and **(B)** interval between the onset of the EMG activity of the submental/suprahyoid muscle complex and the onset of the laryngopharyngeal mechanogram (I-SHEMG-LMP).

The main results are summarized in **Table 2**. Fifty-four of the 67 patients examined (81%) showed a clinical response after the first treatment with BTX: 35 patients (52.2%) were classified as high responders while other 19 patients (28.4%) were low responders based on the DOSS score changes observed at the first 1-month follow-up (**Figures 1** and **2**). The remaining 13 patients (19.4%) were non-responders: 10 patients showed no change in the DOSS score, and 3 patients showed a reduction of the baseline DOSS score.

The beneficial clinical effect of the first treatment lasted more than 4 months (range: 18 to 46 weeks) in 36 of the 54 responders (67%), between 2 and 4 months (range: 8–16 weeks) in 16 (30%) and less than 2 months (range: 6–7 weeks) in the remaining two (4%) (see **Table 3** for more details). The chi-square test showed that the duration of the beneficial effects of the BTX treatment differed between diseases, being longer in patients with post-traumatic encephalopathy and shorter in parkinsonian syndromes (χ^2^= 8.22, df = 3, *p* = 0.043).

**Table 3:** Duration of the effects of first BTX injection in responders with neurogenic dysphagia.

	Total	Parkinsonian syndrome	Stroke	Post-traumatic encephalopathy	Multiple Sclerosis
Number of cases	54	16	19	10	9
<2months	2 (3.7%)	2 (12.5%)	0 (0.0%)	0 (0.0%)	0 (0.0%)
2–4 months	16 (29.6%)	6 (37.5%)	4 (21.1%)	1 (10.0%)	5 (55.6%)
>4 months	36 (66.7%)	8 (50.0%)	15 (78.9%)	9 (90.0%)	4 (44.4%)

*Differences between neurological disorders: χ^2^= 8.22, df = 3, *p* = 0.043.*

With regard to the electrophysiological assessment, five of the 13 patients classified as non-responders (38.5%) had SHEMG-D values that fell below the normal range, in one case associated with an increase in the I-SHEMG-LMP; three patients (23.1%) showed increased SHEMG-D and I-SHEMG-LMP values; two (15.4%) showed an abnormal increase only in the I-SHEMG-LMP parameter. Three of the 13 non-responders (23.1%) showed normal values both for SHEMG-D and for I-SHEMG-LMP.

The SHEMG-D value was above the normal limit in four of the 36 high responders (11.1%) and three of the 18 low responders (16.7%), while the I-SHEMG-LMP value was above the normal limit in only five of the high responders (13.9%) and two of the low-responders (11.1%).

The I-SHEMG-LMP parameter was statistically higher in the non-responders compared with the high responders (*Z* = –2.49, *p* = 0.01). Indeed, no significant differences were observed between high responders and low responders or between low responders and non-responders (**Figure 2B**). The SHEMG-D parameter did not show significant differences between high responders, low responders and non-responders to BTX treatment (**Figure 2A**). This was probably due to the high variability of this parameter in the group of non-responders, in which both abnormally increased and decreased values were observed.

All the patients received the second BTX treatment as we did not observe any severe side effects after the first BTX injection. Fifty-three of the 67 patients examined (79%) were responsive to the second treatment with BTX: 31 patients (46.3%) showed a high response, 22 patients (32.8%) a low response, and the remaining 14 patients (20.9 %) were non-responsive to the treatment (see **Table 2** for more details).

The chi-square test revealed a significant difference in the distribution of high/low responders and non-responders between the first and the second BTX treatment with a reduction in the efficacy of the treatment following the second injection (χ^2^= 16.16, df = 4, *p* = 0.003). This was mainly due to a reduction of efficacy in the group of patients with parkinsonian syndromes in which the percentage of high responders fell from 42.8% (9 out of 21 patients) to 9.5 % (2 out of 21 patients).

## Discussion

The results of our study confirm the efficacy of BTX injection for the treatment of neurogenic dysphagia associated with hyperactivity of the CP muscle. As shown by the DOSS score changes after the first BTX treatment, dysphagia improved in more than 80% of patients with beneficial effects that lasted over 4 months in most of them. In agreement with previous findings of other groups, in some cases the efficacy of the treatment lasted up to one year (Ahsan et al., 2000; Haapaniemi et al., 2001; Shaw and Searl, 2001; Zaninotto et al., 2004; Terré et al., 2008, 2013; Woisard-Bassols et al., 2013). Overall, our results are in line with the findings reported in a review of the literature, showing that the mean success rate of BTX in the management of dysphagia linked to dysfunction of the CP muscle is of 76%, similar to that of myotomy (75%) (Kocdor et al., 2016). However, it is noteworthy that the success rates reported in the different studies may vary between 43 and 100%. This is likely due to different variables including patients’ selection, technical aspects (e.g., injection of BTX under endoscopy or EMG guidance), and injection sites and total dose of BTX. For instance, some authors have used a higher BTX dose (100 U) than that used in the present study, and they have injected it in three different sites of the CP muscle under the guidance of a flexible endoscope (Terré et al., 2008, 2013), showing a longer lasting effect (at least 12 months) in the absence of complications.

In the present work, we also showed that a second BTX injection may result in an improvement of dysphagia in a large proportion of the patients, although the improvement was less marked, especially in the patients with parkinsonian syndromes.

The CP muscle is the main portion of the UES and its activity as a sphincter is fundamental for controlling orthograde and retrograde esophageal flows (Lang and Shaker, 2000; Sivarao and Goyal, 2000). It is important to note that incomplete or absent relaxation of the UES during swallowing does not equate with incomplete or absent opening of this structure. UES opening is the result of a complex interaction between neural control and biomechanical events related to adjacent structures, and consequently its impairment may depend on several factors (Lang and Shaker, 2000). For instance, fibrosis of intrinsic sphincter muscles and persistent muscle tone may result in either diminished or absent UES opening. Furthermore, poor pharyngeal constrictor muscle function can also result in inefficient propulsion of the bolus from the pharynx to the esophagus. These abnormalities, combined with absent or incomplete opening of the UES during swallowing, can lead to obstruction of bolus transit from the pharynx to the esophagus and increase the likelihood of a post-deglutition aspiration of food (Lang and Shaker, 2000; Sivarao and Goyal, 2000).

Although opening and closure of the UES during swallowing can be indirectly documented by means of FEES (Horner et al., 1993; Doggett et al., 2001), needle EMG is able to directly evaluate changes in the activity of the UES muscle, thus providing fundamental information on the pathophysiological mechanisms underlying dysphagia (Alfonsi et al., 2010). Therefore, conventional swallowing investigations should always be supplemented by an EMG study for guiding the decision to inject or not BTX into the CP muscle in patients with dysphagia.

We have previously reported that electrophysiological investigations can reveal restoration of the normal EMG-CP-pause after the BTX treatment (Alfonsi et al., 2010). In this study, we confirm our previous finding that abnormal I-SHEMG-LMP and/or SHEMG-D values can predict a negative response to this BTX treatment (Alfonsi et al., 2010). However, in the present study, we also found that a small proportion of patients with abnormal I-SHEMG-LPM and/or SHEMG-D values responded positively to the treatment. Although this indicates that electrophysiological findings can help in selecting which patients to treat, ultimately the decision in an individual patient should not be taken only on the basis of the presence or absence of electrophysiological abnormalities. Indeed, in some cases, suprahyoid/submental muscle dysfunction may be a less important factor in inducing dysphagia than abnormal UES opening. For instance, in patients with camptocormic postures and neck flexion, the synergistic action of other muscles, such as the infrahyoid muscles, could compensate for the bradykinesia/hypokinesia of the suprahyoid/submental muscle complex (Lang and Shaker, 2000) that may be responsible for increased SHEMG-D values.

As regards, safety of the BTX treatment, our findings are in line with the complication rates reported in the literature, which range from 0 to 25% (Kocdor et al., 2016). Indeed, in 15% of our patients (all non-responders), the first BTX injection into the CP muscle was associated with mild side effects, namely transient worsening of the dysphagia or the appearance of dysphonia (whispering and breathiness). These side effects were likely related to the inadvertent diffusion of BTX to nearby muscles. Diffusion of BTX from the injection site to muscles situated above it, such as the inferior constrictor muscle of the pharynx, may indeed result in impaired pharyngeal peristalsis, while its diffusion to nearby tensor and abductor muscles of the vocal cords may result in voice and breathing abnormalities. These side effects might be limited by reducing the BTX dilution (Alfonsi et al., 2010). Conversely, it is possible that in the non-responders who did not manifest side-effects might have benefitted from higher doses of the toxin.

Though after the second BTX injection we recorded a lower rate of adverse events (10%), a severe side effect was observed in two patients (3%) who were both non-responders to the first BTX injection and had undergone the second treatment after 1 month. This suggests caution in the repetition of treatment in non-responders. The choice to repeat the treatment should be carefully weighed against the risks, possibly considering a longer time interval from the first administration.

Our finding that side effects and complications of the BTX injection occurred only in non-responders both after the first and the second treatment deserves to be discussed. It may be that, in some cases, the UES hypertonia was essentially a protective mechanism, serving to prevent reflux of material from the esophagus into the pharynx (Shaker and Hogan, 2000). This mechanism might be observed in patients with gastroesophageal reflux, however, this specific aspect was not investigated in the present study.

In our dysphagic patients with post-traumatic encephalopathy, the effect of the BTX treatment was more marked after the first injection, but they also showed an excellent response to the second treatment. Since all these patients had a marked spastic-dystonic syndrome, it is possible that muscle hyperactivity involving not only the UES but also other neighboring muscles was the major pathophysiological mechanism underlying the dysphagia in this group. It follows that in these patients BTX injection could result in a clinical improvement even were the toxin to diffuse to the pharyngeal-laryngeal muscles contiguous to the CP muscle.

On the contrary, in the patients with parkinsonian syndromes the effect of the treatment tended to decrease over time and to be less effective when repeated. There may be different reasons for this. First, in degenerative disorders, declining BTX efficacy could be due to the progressive degeneration of different structures of the central nervous system involved in the swallowing function (Bass and Morrel, 1992; Henderson et al., 2000; Benarroch et al., 2002; Pfeiffer, 2003). Furthermore, the lower efficacy of both the first and second treatments in parkinsonian syndromes may also be due to the impaired cortical plasticity that is a consequence of the dopaminergic deficit characteristic of these syndromes (Eggers et al., 2010; Conte et al., 2012; Udupa and Chen, 2013); as a result of this impaired plasticity, the capacity for modification of swallowing behavior after BTX treatment would be reduced. It is also noteworthy that cortical plasticity worsens during the course of the disease (Udupa and Chen, 2013) and, in this regard, it may be relevant that our patients presented with a relatively long duration of the disease.

The absence of a control group is a critical limitation of this study; however, our finding of marked clinical improvements over a prolonged period in the majority of the patients indicates that biases due to random fluctuations or placebo effects are unlikely. Furthermore, given that the efficacy of BTX for the treatment of neurogenic dysphagia is well documented (Alberty et al., 2000; Ahsan et al., 2000; Restivo et al., 2002, 2013; Moerman, 2006; Alfonsi et al., 2010), the use of a no-treatment control group would pose considerable ethical problems.

## Conclusion

The results of this study confirm previous observations that BTX treatment is highly effective in the treatment of neurogenic dysphagia due to hyperactivity and relaxation failure of the UES. EES may provide useful information regarding the different pathophysiological mechanisms at work in different kinds of neurogenic dysphagia, thus improving the selection of patients more likely to benefit from the BTX treatment and less likely to experience its potential adverse effects. Finally, in our view, an important “caveat” of BTX treatment is that absence of a therapeutic response to the first injection should be considered a risk factor for side possible effects (for which patients should be closely and carefully followed up); on this basis, it should also be taken as an indication not to repeat the treatment.

## Author Contributions

Substantial contributions to the conception or design of the work; or the acquisition, analysis, or interpretation of data for the work: EA, GC. Drafting the work or revising it critically for important intellectual content: EA, DR, CG, RDI, GB, AS, MT, MF, AC, PP, MR, AM, GS, CT. Final approval of the version to be published: EA, DR, CG, RDI, GB, AS, MT, MF, AC, PP, MR, AM, GS, CT. Agreement to be accountable for all aspects of the work in ensuring that questions related to the accuracy or integrity of any part of the work are appropriately investigated and resolved: EA, DR, CG, RDI, GB, AS, MT, MF, AC, PP, MR, AM, GS, CT.

## Conflict of Interest Statement

The authors declare that the research was conducted in the absence of any commercial or financial relationships that could be construed as a potential conflict of interest.

## References

[B1] Ahsan S. F. Meleca R. J. Dworkin J. P. (2000). Botulinum toxin injection of the cricopharyngeus muscle for the treatment of dysphagia. *Otolaryngol. Head Neck. Surg.* 122 691–695. 10.1016/s0194-5998(00)70198-7 10793348

[B2] Alberty J. Oelerich M. Ludwig K. Hartmann S. Stoll S. (2000). Efficacy of botulinum toxin A for treatment of upper esophageal sphincter dysfunction. *Laryngoscope* 110 1151–1156. 10.1097/00005537-200007000-00016 10892687

[B3] Alfonsi E. Bergamaschi R. Cosentino G. Ponzio M. Montomoli C. Restivo D. A. (2013). Electrophysiological patterns of oropharyngeal swallowing in multiple sclerosis. *Clin. Neurophysiol.* 124 1638–1645. 10.1016/j.clinph.2013.03.003 23601703

[B4] Alfonsi E. Merlo I. M. Ponzio M. Montomoli C. Tassorelli C. Biancardi C. (2010). An electrophysiological approach to the diagnosis of neurogenic dysphagia: implications for botulinum toxin treatment. *J. Neurol. Neurosurg. Psychiatry* 81 54–60. 10.1136/jnnp.2009.174698 19762326

[B5] Alfonsi E. Versino M. Merlo I. M. Pacchetti C. Martignoni E. Bertino G. (2007). Electrophysiologic patterns of oral-pharyngeal swallowing in parkinsonian syndromes. *Neurology* 68 583–589. 10.1212/01.wnl.0000254478.46278.67 17310027

[B6] Bass N. H. Morrel R. M. (1992). “The neurology of swallowing,” in *Dysphagia. Diagnosis and Management* 2nd Edn ed. Groher M. E. (Boston: Butterworth-Heinemann) 1–30.

[B7] Benarroch E. E. Schmeichel A. M. Parisi J. E. (2002). Depletion of mesopontine cholinergic and sparing of raphe neurons in multiple system atrophy. *Neurology* 59 944–946. 10.1212/WNL.59.6.944 12297588

[B8] Conte A. Belvisi D. Bologna M. Ottaviani D. Fabbrini G. Colosimo C. (2012). Abnormal cortical synaptic plasticity in primary motor area in progressive supranuclear palsy. *Cereb. Cortex* 22 693–700. 10.1093/cercor/bhr149 21677027

[B9] Doggett D. L. Tappe K. A. Mitchell M. D. Chapell R. Coates V. Turkelson C. M. (2001). Prevention of pneumonia in elderly stroke patients by systematic diagnosis and treatment of dysphagia: an evidence-based comprehensive analysis of the literature. *Dysphagia* 16 279–295. 10.1007/s00455-001-0087-3 11720404

[B10] Eggers C. Fink G. R. Nowak D. A. (2010). Theta burst stimulation over the primary motor cortex does not induce cortical plasticity in Parkinson’s disease. *J. Neurol.* 257 1669–1674. 10.1007/s00415-010-5597-1 20496035

[B11] Ertekin C. Pehlivan M. Aydoğdu I. Ertas M. Uludağ B. Celebi G. (1995). An electrophysiological investigation of deglutition in man. *Muscle Nerve* 18 1177–1186. 10.1002/mus.880181014 7659112

[B12] Gilman S. Wenning G. K. Low P. A. Brooks D. J. Mathias C. J. Trojanowski J. Q. (2008). Second consensus statement on the diagnosis of multiple system atrophy. *Neurology* 71 670–676. 10.1212/01.wnl.0000324625.00404.15 18725592 PMC2676993

[B13] Haapaniemi J. J. Laurikainen E. A. Pulkkinen J. Marttila R. J. (2001). Botulinum toxin in the treatment of cricopharyngeal dysphagia. Review of the use of botulinum in treating proximal dysphagia. *Dysphagia* 16 171–175. 10.1007/s00455-001-0059-7 11453562

[B14] Henderson J. M. Carpenter K. Cartwright H. Halliday G. M. (2000). Loss of thalamic intralaminar nuclei in progressive supranuclear palsy and Parkinson’s disease: clinical and therapeutic implications. *Brain* 123 1410–1421. 10.1093/brain/123.7.1410 10869053

[B15] Hiss S. G. Postma G. N. (2003). Fiberoptic endoscopic evaluation of swallowing. *Laryngoscope* 113 1386–1393. 10.1097/00005537-200308000-00023 12897564

[B16] Horner J. Brazer S. R. Massey E. W. (1993). Aspiration in bilateral stroke patients: a validation study. *Neurology* 43 430–433. 10.1212/WNL.43.2.430 8437716

[B17] Hughes A. J. Daniel S. E. Kilford L. Lees A. J. (1992). Accuracy of clinical diagnosis of idiopathic Parkinson’s disease: a clinico-pathological study of 100 cases. *J. Neurol. Neurosurg. Psychiatry* 55 181–184. 10.1136/jnnp.55.3.181 1564476 PMC1014720

[B18] Kaas J. H. Merzenich M. M. Killackey H. P. (1983). The reorganization of somatosensory cortex following peripheral nerve damage in adult and developing mammals. *Annu. Rev. Neurosci.* 6 325–356. 10.1146/annurev.ne.06.030183.001545 6340591

[B19] Kocdor P. Siegel E. R. Tulunay-Ugur O. E. (2016). Cricopharyngeal dysfunction: a systematic review comparing outcomes of dilatation, botulinum toxin injection, and myotomy. *Laryngoscope* 126 135–141. 10.1002/lary.25447 26360122

[B20] Krause E. Schirra J. Gurkov R. (2008). Botulinum toxin A treatment of cricopharyngeal dysphagia after subarachnoid haemorrage. *Dysphagia* 23 406–410. 10.1007/s00455-007-9132-1 18437465

[B21] Lang I. M. Shaker R. (2000). An overview of the upper esophageal sphincter. *Curr. Gastroenterol. Rep.* 2 185–190. 10.1007/s11894-000-0059-z 10957928

[B22] Litvan I. Agid Y. Calne D. Campbell G. Dubois B. Duvoisin R. C. (1996). Clinical research criteria for the diagnosis of progressive supranuclear palsy (Steele-Richardson-Olszewski syndrome): report of the NINDS-SPSP international workshop. *Neurology* 47 1–9. 10.1212/WNL.47.1.1 8710059

[B23] McDonald W. I. Compston A. Edan G. Goodkin D. Hartung H. P. Lublin F. D. (2001). Recommended diagnostic criteria for multiple sclerosis: guidelines from the International Panel on the diagnosis of multiple sclerosis. *Ann. Neurol.* 50 121–127. 10.1002/ana.1032 11456302

[B24] Moerman M. B. (2006). Cricopharyngeal botox injection: indication and technique. *Curr. Opin. Otolaryngol. Head Neck Surg.* 14 431–436. 10.1097/MOO.0b013e328010b85b 17099352

[B25] O’Neil K. H. Purdy M. Falk J. Gallo L. (1999). The dysphagia outcome and severity scale. *Dysphagia* 14 139–145. 10.1007/PL00009595 10341109

[B26] Orsini M. Leite M. A. Chung T. M. Bocca W. de Souza J. A. de Souza O. G. (2015). Botulinum neurotoxin type A in neurology: update. *Neurol. Int.* 7:5886 10.4081/ni.2015.5886 PMC4591494 26487928

[B27] Pfeiffer R. F. (2003). Gastrointestinal dysfunction in Parkinson’s disease. *Lancet Neurol.* 2 107–116. 10.1016/S1474-4422(03)00307-7 12849267

[B28] Regan J. Murphy A. Chiang M. McMahon B. P. Coughlan T. Walshe M. (2014). Botulinum toxin for upper oesophageal sphincter dysfunction in neurological swallowing disorders (Review). *Cochrane Database Syst. Rev* 5 CD009968 10.1002/14651858.CD009968.pub2 PMC10600350 24801118

[B29] Restivo D. A. Casabona A. Nicotra A. Zappia M. Elia M. Romano M. C. (2013). ALS dysphagia pathophysiology: differential botulinum toxin response. *Neurology* 80 616–620. 10.1212/WNL.0b013e318281cc1b 23345638

[B30] Restivo D. A. Palmeri A. Marchese-Ragona R. (2002). Botulinum toxin for cricopharyngeal dysfunction in Parkinson’s disease. *N. Engl. J. Med.* 346 1174–1175. 10.1056/NEJM200204113461517 11948283

[B31] Shaker R. Hogan W. J. (2000). Reflex-mediated enhancement of airway protective mechanisms. *Am. J. Med.* 108(Suppl. 4a) 8S–14S. 10.1016/S0002-9343(99)00289-2 10718445

[B32] Shaw G. Y. Searl J. P. (2001). Botulinum toxin treatment for cricopharyngeal dysfunction. *Dysphagia* 16 161–167. 10.1007/s00455-001-0074-8 11453560

[B33] Sivaraman Nair K. P. (2016). Review of Manual of botulinum toxin therapy, 2nd ed. *JAMA Neurol.* 73 359.

[B34] Sivarao D. V. Goyal R. K. (2000). Functional anatomy and physiology of the upper esophageal sphincter. *Am. J. Med.* 108(Suppl. 4a) 27S–37S. 10.1016/s0002-9343(99)00337-x 10718448

[B35] Terré R. Panadés A. Mearin F. (2013). Botulinum toxin treatment for oropharyngeal dysphagia in patients with stroke. *Neurogastroenterol. Motil.* 25 896-e702 10.1111/nmo.12213 23991889

[B36] Terré R. Valles M. Panades A. Mearin F. (2008). Long-lasting effect of a single botulinum toxin injection in the treatment of oropharyngeal dysphagia secondary to upper esophageal sphincter dysfunction: a pilot study. *Scand. J. Gastroenterol.* 43 1296–1303. 10.1080/00365520802245403 18649151

[B37] Udupa K. Chen R. (2013). Motor cortical plasticity in Parkinson’s disease. *Front. Neurol.* 4:128 10.1038/tp.2014.125 PMC3761292 24027555

[B38] Woisard-Bassols V. Alshehri S. Simonetta-Moreau M. (2013). The effects of botulinum toxin injections into the cricopharyngeus muscle of patients with cricopharyngeus dysfunction associated with pharyngo-laryngeal weakness. *Eur. Arch. Otorhinolaryngol.* 270 805–815. 10.1007/s00405-012-2114-4 22865104

[B39] Zaninotto G. Marchese Ragona R. Briani C. Costantini M. Rizzetto C. Portale G. (2004). The role of botulinum toxin injection and upper esophageal sphincter myotomy in treating oropharyngeal dysphagia. *J. Gastrointest. Surg.* 8 997–1006. 10.1016/j.gassur.2004.09.037 15585387

